# Vitamin E and telmisartan attenuates doxorubicin induced cardiac injury in rat through down regulation of inflammatory response

**DOI:** 10.1186/1471-2261-12-63

**Published:** 2012-08-06

**Authors:** Najah Hadi, Nasser Ghaly Yousif, Fadhil G Al-amran, Nadhem K Huntei, Bassim I Mohammad, Sadiq J Ali

**Affiliations:** 1 University of Colorado Denver, department of Medicine and Surgery Aurora, Denver, CO, 80045, USA; 2 Kufa University, College of medicine, Najaf, Iraq; 3 Al-Qadesiyah college of Medicine, Al-Qadesiya, Iraq

## Abstract

**Background:**

The importance of doxorubicin (Dox), as a potent antitumor antibiotic, is limited by the development of life-threatening cardiomyopathy. It has been shown that free radicals are involved in acute doxorubicin-induced toxicity. The aim of this study was to determine the protective effect of vitamin E and telmisartan in acute doxorubicin induced cardiotoxicity.

**Methods:**

Thirty two male Sprague - Dawly rats were involved in this study and were randomly separated into 4 groups, eight rats in each group, one group received normal saline I.P as control and second group received doxorubicin 20 mg/kg I.P, the other two groups also received doxorubicin 20 mg/kg I.P as single dose after seven cumulative doses (for seven days) of vitamin E (100 mg/kg) and telmisartan (1 mg/kg) respectively. Immunofluorescent staining for monocytes infiltration and analyses of plasma by (ELISAs) for MCP-1and troponin I. Western immunoblotting assay for ICAM-1, while left ventricular function was analyzed by microcatheter, also estimated the level of oxidative stress parameters (MDA and Catalase) and cardiac enzymes activities (CK-MB and LDH) before starting drugs treatment and after treatment period by 48 hours.

**Results:**

The immunofluorescent staining showed that administration of vitamin E and telmisartan are attenuated of mononuclear cell infiltration; (p < 0.05 vs. Dox group), also reduced the level of chemokines MCP-1 and ICAM-1 expression compared with Dox group only, and there is marked reduction of myocardial troponin-I levels with improved LV function in vitamin E and telmisartan treated group. Doxorubicin treatment increased MDA, LDH, CK-MB levels significantly (P < 0.01), and were counteracted by administration of vitamin E and telmisartan, but did not significantly affect serum catalase activity.

**Conclusions:**

Antioxidant effect (Vitamin E and telmisartan) have been shown to decrease doxorubicininduced cardiotoxicity.

## Background

Doxorubicin (Dox) is a cytotoxic anthracycline antibiotic isolated from cultures of Streptomyces peucetius var. caesius, and is commonly used in the treatment of a wide range of cancers, including hematological malignancies, many types of carcinoma, and soft tissue sarcomas [1]. A number of different mechanisms have been proposed for the antitumor action of Dox these include doxorubicin interacts with DNA by intercalation and inhibition of macromolecular biosynthesis [2,3], this inhibits the progression of the enzyme topoisomerase II, which relaxes supercoils in DNA for transcription, Dox stabilizes the topoisomerase II complex after it has broken the DNA chain for replication, and preventing the DNA double helix from being resealed and thereby stopping the process of replication [4].

The therapeutic value of Dox as anticancer antibiotic is limited by its acute as well as accumulative dose related cardiotoxicity and by 1967, it was recognized that Dox could produce fatal cardiac toxicity [5], currently, there is no specific therapeutic strategy against this severe disease, and cardiac transplantation remains a vital option for patients with end-stage Dox-induced heart failure [6]. The pathogenesis of Dox-induced cardiotoxicity and heart failure is complex and may involve various signaling mechanisms including free radical stress, calcium overloading, mitochondrial dysfunction dysregulation of iron haemostasis [7], alteration in beta-adrenergic receptor signaling [8], mitochondrial dysfunction [9], and activation of matrix metalloproteinase [10]. Also it is notable that, cytokine release mediated by activation of the innate immune system is believed to be involved in the pathogenesis of Dox-induced cardiotoxicity [11], the Free radicals and other toxic non-radicals generated by anthracyclines can be neutralized by increasing endogenous antioxidants or by introducing exogenous antioxidants through nutritional supplements, these interventions may prevent or attenuate the side-effects of anthracyclines these anti-oxidants, including vitamins especially (Vitamin A and provitamin A carotenoids, Vitamin C, Vitamin E) coenzyme Q and carnitine can be derived from the diet. In general, these compounds do not interfere with anthracycline activity in tumor cells.

Our hypothesis is focus on the effective role of vitamin E and telmisartan in attenuated doxorubicin induced cardiotoxicity in rat through down regulation of inflammatory response.

## Methods

Male Sprague - Dawly rats, acclimatized in a quarantine room for 2 weeks, and all experiments were approved by the Animal Care and Research Committee of the University of Colorado Denver, and this investigation conforms to the Guide for the Care and Use of Laboratory Animals (National Research Council, revised 1996). The animals divided in to 4 groups, eight rats in each group, control groups (normal saline injected I.P) and group treated with 20 mg/kg doxorubicin in a single injection intraperitoneal i.p. and other two groups one received vitamin E (100 mg/kg) [12] orally by N/G tube for one week followed by doxorubicin (20 mg/kg) I.P as single dose, and the other one received telmisartan (1 mg/kg) [13] orally by N/G tube for one week followed by doxorubicin (20 mg/kg) I.P as single dose.

### Left ventricular function analysis

Pressure-volume loop and hemodynamic analysis was planned for all animals as described previously by N.Yousif,2011 [14], in briefly the animals anesthetized with ketamin in dose of 50 mg/kg injected intraperitoneal and neck was opened longitudinally and right common carotid artery exposed and freed, ligated distally and stay suture placed proximally, then small opening was made in artery and size 1 F-micro tipped pressure transducer catheter (Millar Instruments, Houston, TX, USA) was inserted into the LV lumen via the right carotid artery for measurement of LV pressure, volume, function and related parameters. Then after about 20 minutes of data recording, the abdomen is opened by right sub costal incision to reach the inferior vena cava. To acutely change the cardiac preload, caval occlusion was produced over a 3-s period using a nonmetallic occluder applied to the IVC. The data were recorded as a series of pressure-volume loops.

P van software (Conductance Technologies, San Antonio, TX, and Millar, Houston, TX) was used to analyze all pressure-volume loop data. Regression analyses of multiple isochronal pressure-volume loop data were produced by IVC compression. From the baseline and IVC compression loops, comprehensive sets of hemodynamic parameters were calculated. All steady-state and caval occlusion pressure-volume loops were acquired with the computer data acquisition system. From these data we selected the following parameter: LV diameters were measured at end diastole (LVEDD) and end systole (LVESD), ejection fraction (EF), heart rate (HR), LV systolic pressure in the ends of both systole and diastole (LVESP, LVEDP), Cardiac Output (CO) and the maximum elasticity (Emax).

### Animal scarification

After complete assessed the pressure volume loop, the animal was sacrificed, starting by injection of equal volume of thiopental and heparin intraperotonealy in doses ranging from 300 μl to 600 μl for each one, after giving good time for the animal to go into deep anesthesia, the rat is positioned and taped and the chest is opened in flap like manner revealing the heart then a needle of the syringe is introduced into right ventricle to aspirate around 0.5 ml of blood for plasma analysis.

### Immunofluorescent staining

Immunofluorescent staining was performed as described previously [14]. Myocardial cryosections (5 μm thick) were treated with a mixture of 30% methanol and 70% acetone and fixed in 4% paraformaldehyde. Sections were incubated with blocking solution (10% BSA in PBS) for 45 min. Sections were then incubated with a mixture of rat monoclonal antibody against mouse neutrophil marker protein (Clone 7/4; ABD Serotec, Oxford, UK) and rabbit polyclonal antibody against mouse macrophage marker protein CD68 (Santa Cruz Biotechnology, Santa Cruz, CA), 5 μg/ml each in antibody buffer, for 90 min. Sections were then incubated for 45 min with a mixture of Cy3-labeled goat anti-rat IgG (labeling neutrophils red; 1:250 dilution with antibody buffer) and Alexa488-conjugated goat anti-rabbit IgG (labeling macrophages green; 1:400 dilution with antibody buffer). Sections were counterstained with Alexa Fluor 350-labeled wheat germ agglutinin (for outlining myocardial cells). To assess the staining specificity, adjacent sections were incubated with a mixture of nonimmune rat IgG and nonimmune rabbit IgG (5 μg/ml each in antibody buffer) in replacement of the primary antibodies and then processed in identical conditions. Microscopic observation and photography were performed with a Leica DMRXA digital microscope. Images were analyzed using Slide Book 2.6 software to obtain quantitative estimates of area.

### Blood sample

The collected blood from each rat was centrifuged (in 10000 RPM, for 10 minutes at 4 °C) and the yielded plasma samples was obtained before starting drug treatment (at zero time) and after 48 hours of completion treatment (i.e. after 9 days) for determination of serum malondialdehyde (MDA) Level (the byproduct of lipid peroxidation); Serum Catalase Activity (CAT enzyme); Serum Creatine kinase (CK-MB) enzyme level and Serum Lactate dehydrogenase (LDH) enzyme level.

### Enzyme-linked immunosorbent assays (ELISAs)

Myocardial MCP-1 and troponin I were measured using commercially available ELISA kits (R and D Systems) according to the manufacturer's instructions.

### Western immunoblotting assay

Myocardial tissue was homogenized with a rotor-stator homogenizer and treated in PBS containing 0.5% Triton X-100 and a protease inhibitor cocktail. Size fraction of crude protein (20 μg) was performed by electrophoresis. After transfer, the membrane was incubated in PBS 5% nonfat dry milk to block nonspecific binding. The membrane was then incubated for 60 minutes with an antibody against ICAM-1, at 1:1000 to 1:2000 dilutions with PBS containing 0.05% Tween 20 and 5% dry milk. After thorough washes, the membrane was treated with peroxidase-labeled secondary antibody (1:5000 dilutions with phosphate-buffered saline containing 0.05% Tween 20 and 5% dry milk) for 45 minutes. Protein bands were developed using enhanced chemiluminescence technique. Densitometry was performed using a computerized densitometer (Molecular Dynamics, Sunnyvale, CA).

### Statistical analysis

The data were expressed as mean +/− standard deviation (SD) were calculated and compared using ANOVA test. Significance was set at p < 0.05 for all comparisons unless otherwise stated. Statistical analysis has been done by using paired t-test and ANOVA test. Significant difference was set at P < 0.05 [15].

## Results

### Vitamin E and telmisartan improves LV function

To determine the degree of heart injury after Dox injection with and without antioxidant agents, we measured LV function in vivo by assessing pressure-volume loops using a micro-conductance catheter. Hemodynamic data are shown in (Table 1). Dox injected only group show displayed impaired systolic and diastolic (LVP, LVEDP, CO), and antioxident agent treated group show improved in the LV function.

**Table 1:** Selected average parameters from pressure-volume loop study in vivo assessment of left ventricular function are expressed as mean ± SEM

**Variables**	**Control**	**Dox**	**Vitamin E Telmisartan**	**P value**
End-systolic Volume (uL)	43.64 ± 1.23	69.61 ± 2.36	51.34 ± 1.22	<0.05
End-diastolic Volume (uL)	69.61 ± 2.37	85.03 ± 3.53	63.21 ± 1.43	<0.05
End-systolic Pressure (mmHg)	70.57 ± 1.22	56.64 ± 6.76	64.65 ± 1.75	<0.05
End-diastolic Pressure (mmHg)	8.36 ± 1.19	3.42 ± 0.36	7.14 ± 1.54	<0.05
Ejection Fraction (%)	42.34 ± 3.21	17.35 ± 0.57	25.43 ± 1.21	<0.05
Cardiac Output (ml/min)	12.67 ± 1.26	6.03 ± 0.4	7.83 ± 1.17	<0.51
E MAX	21.38 ± 2.36	14.34 ± 1.32	16.43 ± 1.	<0.51

P < 0.05, statistically differences between Dox treated only group and with Vitamin E, and Telmisartan treatment; (n = 8 per group).

### Vitamin E, and telmisartan have a reduced myocardial macrophages accumulation

**Figure 1:**
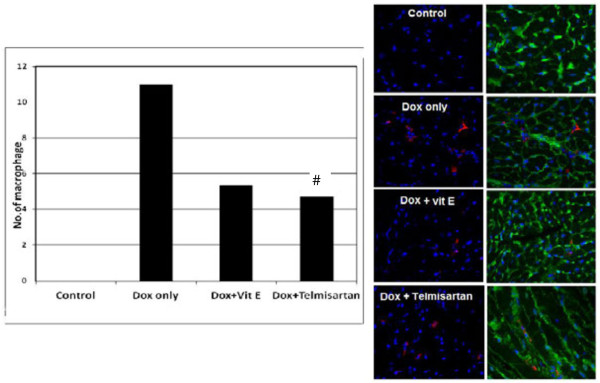
**Immunofluorescent staining demonstrates that Dox treatment only group of rat exhibit greater macrophages infiltration than vitamin E and telmisartan group.** Data are expressed as mean ± SEM. ^#^P < 0.05; vs. Dox only group (n = 8 per group).

Dox only treated rat group displayed significantly enhanced infiltration of macrophages compared to antioxident agent treated rat group as in Figure 1.

### Reduced level of cytokines MCP-1 and ICAM-1 expression in vitamin E, and telmisartan treated group

**Figure 2:**
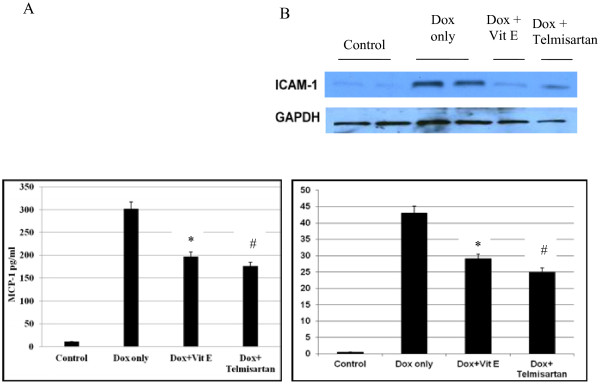
**Dysfunctions of the coronary endothelium as well as injury to cardiac muscle cells are the consequences of Dox effect among many factors, MCP-1and ICAM-1 expression are released into the myocardium and circulation.** Data are expressed as mean ± SEM, (**A**) MCP-1 ^#^*P < 0.05; vs. Dox only (n = 8 per group). (**B**) While the representative western blots showing increased ICAM-1 protein expression in Dox only group ^#^*P < 0.05; vs. Dox only (n = 8 per group).

To characterize the cardiac inflammatory response due to the myocardial toxicity by doxorubicin injection, we measured cardiac chemokines (MCP-1 and ICAM-1), that showed an increased expression of them in the group treated with doxorubicin only compared to Vitamin E, and Telmisartan as in Figure 2.

### Marked decreased in troponin-I levels in vitamin E, and telmisartan treated group

**Figure 3:**
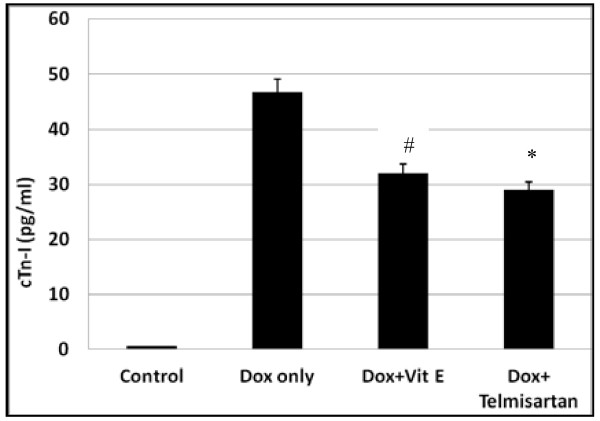
**Cardiac troponin I is very sensitive and specific indicators of damage to the heart muscle (myocardium).** in this figure the level of troponin I is significantly reduction in group of Dox + antioxidants agents; Data are expressed as mean ± SEM, ^#^*P < 0.05; vs. Dox only only (n = 8 per group).

Cardiac troponin I is indicators of cardiac injury, raised troponin level are prognostically important in many of the conditions in which they are used for diagnosis. In present study showed significant reduction in Vitamin E, and Telmisartan treated group after a single dose injection of doxorubicin, and this reflected that the cardiac injury is more in the group treated with doxorubicin only, as shown in Figure 3.

### Significant reduction in MDA and CK-MB in group treated with vitamin E and telmisartan

Dox injected result in a significant elevation of MDA from 3.27 ± 0.52 to 14.8 ± 0.45 (P < 0.01) .The calculated % of increment in MDA was 352%. Treatment with vitamin E and Telmisartan have resulted in significant reduction in doxorubicin induced MDA elevation, the percent of change in MDA was 160% and 249.2% respectively compared to 352% with doxorubicin alone. There was statistically significant differences (P < 0.05) in the means of serum CK-MB level between the doxorubicin group and other two groups treated by vitamin E and Telmisartan as shown in Table 2. Dox lead to a significant elevation of CK-MB level from 685.8 ± 115.4 to 4759.1 ± 137.7 with percentage of rise of 594% compared to baseline value. Vitamin E and Telmisartan treatment prevented the elevation in CK-MB level induced by DOX. with percentage of rise of 71.7%, and 189% respectively compared to DOX. percentage of rise of 594%. There was statistically significant differences (P < 0.05) in the means of serum CK-MB level between the doxorubicin group and other two groups treated by Vitamin E, and Telmisartan as shown in Table 3.

**Table 2:** Effect of doxorubicin, Vitamin E, and Telmisartan on rat serum MDA in μmol/L

**Groups**	**MDA level (μmol/L)**		**Precent of changing**
	**Before treatment**	**After treatment**	
Doxorubicin 15 mg./kg	3.27 ± 0.52	14.8 ± 0.45 ††	352%
Doxo. + Vit E 100 mg./kg	2.98 ± 0.29	7.87 ± 0.99 †† **	164%
Doxo. + Telmisartan1mg/kg.	3.17 ± 0.29	11.1 ± 0.37 †† **	249.2%

The values are expressed as mean ± SEM. † P < 0.05, †† P < 0.01 (means of each group after treatment versus their means before treatment) by using paired t test.

* P < 0.05, ** P < 0.01 (means of Vitamin E, and Telmisartan groups after treatment versus means of Doxorubicin group after treatment) by using LSD test.

**Table 3:** Effect of Doxorubicin, Vitamin E, and Telmisartan on rat serum CK-MB in IU/L

**Groups**	**CK-MB level IU/L**		**Percent of changing**
	**Before treatment**	**After treatment**	
Doxorubicin 15 mg./kg	685.8 ± 115.4	4759.1 ± 137.7 ††	594%
Doxo. + Vit E 100 mg./kg	587.6 ± 116.2	1008.7 ± 181.9 **	71.7%
Doxo. + Telmisartan 1 mg./kg	666.7 ± 146.1	1926.2 ± 155.4 ††**	189%

The values are expressed as mean ± SEM.† P < 0.05, †† P < 0.01 (means of each group after treatment versus their means before treatment) by using paired t test.

* P < 0.05, ** P < 0.01 (means of Vitamin E, and Telmisartan groups after treatment versus means of Doxorubicin group after treatment) by using LSD test.

### Vitamin E, and telmisartan prevented the elevation in LDH level induced by DOX treatment

Dox has resulted in a significant elevation of LDH level from 578.8 ± 72.3 to4328.1 ± 209.5 with percentage of rise of 647% compared to baseline value (P < 0.01). Vitamin E, and Telmisartan prevented the elevation in LDH level induced by Dox treatment with percentage of rise of 155%, and 444% respectively compared to Dox percentage of rise of 647%. There was statistically significant differences (P < 0.05) in the means of serum LDH level between the doxorubicin group and other two groups treated by Vitamin E, and Telmisartan as shown in Table 4.

**Table 4:** Effect of Doxorubicin, Vitamin E, and Telmisartan on rat serum LDH in IU/L

**Groups**	**LDH level IU/L**		**Percent of changing**
	**Before treatment**	**After treatment**	
Doxorubicin 15 mg./kg	578.8 ± 72.3	4328.1 ± 209.5 ††**	647%
Doxo. + Vit E 100 mg./kg	585.1 ± 51.4	1494.9 ± 55.1†† **	155%
Doxo. + Telmisartan 1 mg./kg	570.4 ± 31.9	3108.5 ± 248.9 ††**	444%

The values are expressed as mean ± SEM. † P < 0.05, †† P < 0.01 (means of each group after treatment versus their means before treatment) by using paired t test.

* P < 0.05, ** P < 0.01 (means of Vitamin E, and Telmisartan groups after treatment versus means of Doxorubicin group after treatment) by using LSD test.

### No significance increased in serum catalase level between the doxorubicin group and other two groups treated by vitamin E, and telmisartan

Serum catalase enzyme decreased slightly after treatment in all group, however this reduction did not reach statistical significant differences P > 0.05 in the means of serum catalase level between the doxorubicin group and other two groups treated by Vitamin E, and Telmisartan as shown in Table 5.

**Table 5:** Effect of Doxorubicin, Vitamin E, and Telmisartan on rat serum catalase in IU/L

**Groups**	**Catalase level IU/L**		**Percent of changing**
	**Before treatment**	**After treatment**	
Doxorubicin 15 mg./kg	0.06 ± 0.005	0.045 ± 0.003	-25%
Doxo. + Vit E 100 mg./kg	0.055 ± 0.004	0.047 ± 0.001	-14.5%
Doxo. + Telmisartan 1 mg./kg	0.057 ± 0.002	0.055 ± 0.008	-3.5%

The values are expressed as mean ± SEM. † P < 0.05, †† P < 0.01 (means of each group after treatment versus their means before treatment) by using paired t test.

* P < 0.05, ** P < 0.01 (means of Vitamin E, and Telmisartan groups after treatment versus means of Doxorubicin group after treatment) by using LSD test.

## Discussion

Adriamycin (also called doxorubicin) induces cardiotoxicity through many pathways one of the most accepted mechanisms is through formation of the free radicals. These free radicals cause membrane and macromolecules damage which directly lead to myocardial damage [16]. Under normal physiological conditions, the tissue concentration of FORs is limited due to the existence of a delicate balance between the generation of PRFO and the antioxidant defense system [17]. However, if this balance is disturbed in favor of more PRFO, either through an enhanced production or via a reduction in the endogenous antioxidant defense system or both, the heart is at risk for PRFO-mediated myocardial cell damage. Thus, changes in myocardial antioxidant status and oxidative stress may have profound effects on cardiac structure and function [18]. In the present study, using vitamin E and telmisartan as antioxidant, the hearts of rats treated with Dox only showed increased level of TNF-activation. Furthermore, our data showed reduces in level of MCP-1 and ICAM-1 with antioxidant agent and this decrease in the proinflammatory cytokines and chemokines activity may be caused by the disturbances in the innate immune system mainly in TLR family as described by other [19]. Moreover, in present work, the vitamin E and telmisartan treated group significantly improved LV function compared with Dox injected only group and this confirmed by other study that showed significantly depressed cardiac function in Dox rat model [20]. Cardiac troponin I (cTnI) is a sensitive and specific marker of myocardial injury, the results of the rats injected with Dox and antioxidants agent showed marked reduction in the level of plasma cardiac troponin I and histopathological changes induced by Dox injection only showed more myocardial injury with marked infiltration of monocyte and cellular changes, this coincided with the work of other authors who reported marked granular blood cells in the wide pericapillary space between the disorganized cardiac myocytes [13].

The MDA levels were significantly elevated after a single dose of doxorubicin, this increase in MDA level can be attributed to that the hydroxyl radicals (OH·) greatly enhances the NADH-dependent microsomal lipid peroxidation and thus initiates a lipid radical chain reaction causing oxidative damage to cell membranes. MDA was significantly decreased after vitamin E treatment, this can be explained by that vitamin E allows free radicals to abstract a hydrogen atom from the antioxidant molecule rather than from poly unsaturated fatty acids, thus breaking the chain of free radical reaction; also MDA was significantly decreased after telmisartan treatment, this can be explained by that reactive oxygen species (ROS) are involved in many of the Ang II signaling pathways [21]. LDH activity significantly decreased after antioxidants agent treatment, Przybyszewski et al, 1994, reported that vitamin E diminished the MDA and the activity of LDH and CPK in the serum of gamma irradiated rats due to free radicals scavenging effect, also [22] mention that the reduction in activity of serum LDH in animals treated with telmisartan may be due to the suppression of cardiac injury by AT1 receptor antagonism.

Our study showed that increased significantly in the CK-MB activity after doxorubicin treatment this increase in activity of CK-MB indicates an injury or damage to cardiac cells by doxorubicin which may be due to the inhibition of nucleic acid and protein synthesis [23]. Sridharan & Shyamadevi 2002 [24], attributed the increase of CK-MB to the excessive production of free radicals and lipid peroxides that might have caused leakage of cytosolic enzymes and to membrane cell damage [25,26], and this CK-MB activity decreased after antioxidants treatment also this mention by other study [3,27]Serum catalase level did not significantly changed after doxorubicin, vitamin E, and telmisartan treatment which may be due to that catalase and other antioxidant enzymes other than GPx have been shown to be relatively less sensitive to oxidative stress [28-30].

## Conclusions

Antioxidant effect (Vitamin E, and telmisartan) have been shown to decrease doxorubicin-induced cardiotoxicity as indicated by their ability to decrease the cytokines,MCP-1, ICAM-1and increment in serum MDA level, LDH and CK-MB, and also there is no change in the animals characteristic at the end of the protocol, this experimental work need further investigation to know the exact pathway of antioxidant in attenuated Dox induce cardiac injury.

## Competing interests

The authors declare that they have no competing interest's financial competing interests.

## Authors’ contribution

NGY carried out Left ventricular function analysis, Western immunoblotting assay, Immunofluorescent staining, Enzyme-linked immunosorbent assays (ELISAs) and participated in the design of the study and performed the statistical analysis, participated in the sequence alignment and drafted the manuscript. NH, FGA, NH, BM, SA participated in the design of the study, sequence alignment and drafted the manuscript and performed the statistical analysis. All authors read and approved the final manuscript.

## Pre-publication history

The pre-publication history for this paper can be accessed here:

http://www.biomedcentral.com/1471-2261/12/63/prepub
